# A Personalized 3D-Printed Model for Obtaining Informed Consent Process for Thyroid Surgery: A Randomized Clinical Study Using a Deep Learning Approach with Mesh-Type 3D Modeling

**DOI:** 10.3390/jpm11060574

**Published:** 2021-06-18

**Authors:** Jungirl Seok, Sungmin Yoon, Chang Hwan Ryu, Seok-ki Kim, Junsun Ryu, Yuh-Seog Jung

**Affiliations:** 1 National Cancer Center, Department of Otorhinolaryngology-Head and Neck Surgery, Goyang-si 10408, Korea; junn279@gmail.com (J.S.); changhwanr@ncc.re.kr (C.H.R.); 2 Department of Biomedical Engineering, College of Medicine, Seoul National University, Seoul 03080, Korea; 3 National Cancer Center, Division of Convergence Technology, Goyang-si 10408, Korea; sungmin1551@naver.com; 4 National Cancer Center, Department of Nuclear Medicine, Goyang-si 10408, Korea; skkim@ncc.re.kr

**Keywords:** 3D-printing, anatomic modeling, personalized medicine, thyroidectomy, thyroid cancer, informed consent

## Abstract

The aim of this study was to evaluate the usefulness of a personalized 3D-printed thyroid model that characterizes a patient’s individual thyroid lesion. The randomized controlled prospective clinical trial (KCT0005069) was designed. Fifty-three of these patients undergoing thyroid surgery were randomly assigned to two groups: with or without a 3D-printed model of their thyroid lesion when obtaining informed consent. We used a U-Net-based deep learning architecture and a mesh-type 3D modeling technique to fabricate the personalized 3D model. The mean 3D printing time was 258.9 min, and the mean price for production was USD 4.23 for each patient. The size, location, and anatomical relationship of the tumor and thyroid gland could be effectively presented using the mesh-type 3D modeling technique. The group provided with personalized 3D-printed models showed significant improvement in all four categories (general knowledge, benefits and risks of surgery, and satisfaction; all *p* < 0.05). All patients received a personalized 3D model after surgery and found it helpful to understand the disease, operation, and possible complications and their overall satisfaction (all *p* < 0.05). In conclusion, the personalized 3D-printed thyroid model may be an effective tool for improving a patient’s understanding and satisfaction during the informed consent process.

## 1. Introduction

The term ‘informed consent’ first appeared in a 1957 precedent (Salgo vs. Leland Stanford Junior University Board of Trustees), when the courts helped to establish the role of informed consent in modern medical practice [1]. This legislation requires that a patient be provided information on the benefits and risks by an experienced physician before a medical procedure is performed, which allows patients to actively participate in making decisions and protects physicians from litigation [2]. However, several studies showed that patients lack recall [2,3,4,5] and do not have enough proper knowledge for granting consent [6]. Accordingly, additional time is needed to explain all the details, and an insufficient explanation arising from the time-consuming informed consent process could cause a dispute and interrupt the mutual rapport between physician and patient [7].

To enhance a patient’s understanding, supplementary tools for informed consent have been introduced [8,9]. Recently, 3D-printed phantom models were effectively introduced to obtain informed consent [10,11,12,13]. For example, a common 3D-printed thyroid cancer phantom model was proven to be effective for both a patient’s understanding and a clinician’s explanation [14].

Based on these results, we conducted a randomized prospective study on obtaining informed consent using personalized 3D-printed models of the patients’ thyroid lesions. To this end, we used a deep learning technique for personalized thyroid segmentation and a mesh-type 3D modeling technique previously described [15]. Finally, we evaluated the practical usefulness of this approach to obtain informed consent and analyzed the patient-related factors that influenced the results.

## 2. Materials and Methods

### 2.1. Ethical Considerations

A study protocol for the deep neural network (DNN) modeling was reviewed and approved by the Institutional Review Board (IRB) of the National Cancer Center of the Republic of Korea (No. NCC2019-0206), which granted a waiver of informed consent because the DNN utilized CT series that had been stored without using any personal information. For the randomized prospective study, all participants provided written informed consent according to the policies and procedures approved by the IRB of the same institute (No. NCC2019-0256). The study was registered on cris.nih.go.kr (Identifier: KCT0005069).

### 2.2. The DNN for Thyroid Segmentation

**Figure 1:**
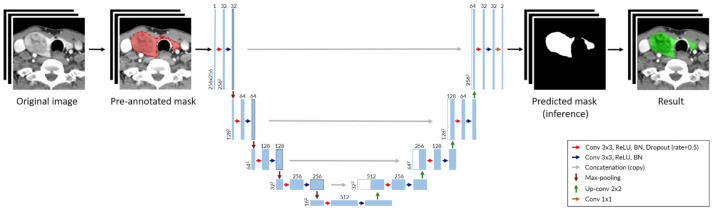
Deep neural network architecture for thyroid segmentation based on U-Net. Each blue box represents a multi-channel feature layer, and each white box is represented as a concatenated layer. The number of channels is shown at the top of boxes and the layer dimensions (x-y size) are denoted at the lower left edge of boxes. (Abbreviation: Conv, convolution layer; BN, batch normalization).

To develop a DNN for thyroid segmentation, we implemented U-Net, the most widely used convolutional neural network (CNN) architecture (Figure 1) [16].

To train the DNN, we collected CT series for 106 consecutive patients who underwent thyroidectomy surgery from January to June 2013. Each CT series was taken using contrast media, according to the ‘Thyroid CT’ protocol. Each series consisted of about 120 images covering the region from the skull base to the thoracic inlet. The images were obtained in Digital Imaging and Communications in Medicine (DICOM) format. The images were transformed into JPEG format, the dimensions of 512 × 512 pixels, resolution of 96 dots per inch (DPI), and depth of 24 bits. The slice thickness was fixed at 2.5 mm, and the pixel spacing was around 0.45 mm per pixel. As the thyroid gland entered the area of 256 × 256 pixels in the center of the image, all images were cropped and used at a size of 256 × 256 pixels throughout the entire process. Since the image is grayscale, it was represented by 1 channel. Therefore, input data of DNN has a 3-dimensional array with lengths of 256, 256, and 1 for each dimension, representing height, width, and number of color channels. The output data is an array of the same size as the input data, which consists of 1 s (predicted mask) and 0 s.

The specific labeling of the thyroid gland area in all CT series was performed by a single experienced thyroid surgeon (JS) using the labelme application (https://github.com/wkentaro/labelme, accessed on 12 March 2020). Another experienced thyroid surgeon (CHR) reviewed and verified the initial labeling. The CT series were randomly divided into three sets (training, validation, and test) at a ratio of 3:1:1, resulting in 63, 21, and 23 series, respectively.

At every training epoch, each slice image of a CT series was augmented randomly using the following methods, with a 70% probability: (1) size scaling from 70% to 130% each width and height; (2) rotation between −10 and +10 degrees; (3) shear between −10 and +10 degrees; (4) coarse noise addition (coarse dropout) with a probability value of 5–10% per pixel over the entire 10–20% of the image area. Finally, a horizontal flip was performed with a 50% probability.

The Dice similarity coefficient (*DSC*) was used as an outcome indicator and defined as:

(1) $$DSC\;\left(y,y^{\prime }\right)=\frac{2\;\left|\;y\;\cap y^{\prime }\;\right|}{\left|\;y\;\right|\cup \left|\;y^{\prime \;}\right|}$$

where y represents the ground-truth, and $y^{\prime }$ is the predicted mask.

Accordingly, the loss function was defined as:

(2) $$L^{DICE}=1-DSC\;\left(y,\;y^{\prime }\right)=1-\frac{2\;\left|\;y\;\cap y^{\prime }\;\right|}{\left|\;y\;\right|\cup \left|\;y^{\prime \;}\right|}$$

The batch size was set to 16 images, and training was performed using Titan V (NVIDIA Co., Santa Clara, CA, USA) as a graphic processing unit in a personal computer.

For evaluating our DNN during the prospective study, post-processing for adding or removing wrongly segmented areas was calculated. DSCs of the enrolled patients were also collected. In contrast to the training, there was no pre-annotated ground truth in the prospective study. As a result, the area after correction of the inference by the DNN was regarded as the ground truth. Accordingly, if the inference was not changed, the DCS was calculated as 1.000. The correction of the inference by the DNN of all CT series was made by an experienced physician (JS).

### 2.3. Development of an Application Incorporating the DNN for 3D Thyroid Reconstruction

We developed a graphic user interface (GUI) based application incorporated with the trained DNN and added a function to correct the area our DNN inferred wrongly. In addition, the application enabled clinicians to mark thyroid nodules on CT images. As not every nodule had clinical significance for decision making, only lesions that could affect surgical extent were expressed by allowing the physician to mark lesions manually.

**Figure 2:**
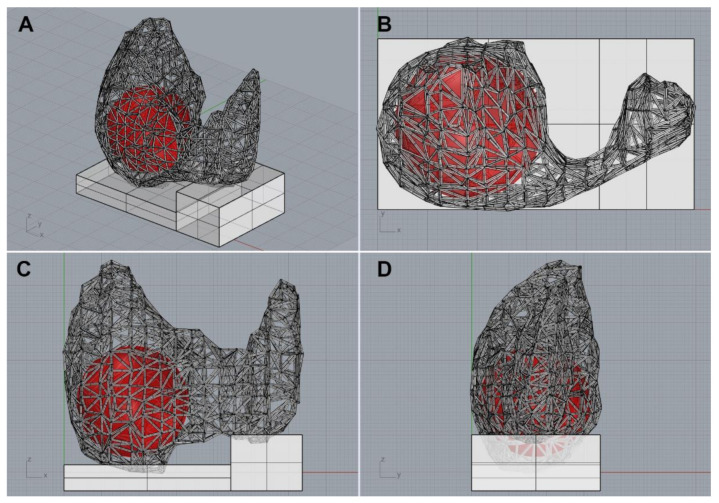
A 3D thyroid model imported from the 3D modeling software. (**A**) Diagonal view, (**B**) top view, (**C**) front view, (**D**) side view.

Using the application, physicians could easily execute DNN segmentation, modify the inferred area manually, mark thyroid nodules with a circle, and export to files (Video S1 in the supplement). After completing the segmentation and marking, the data was reconstructed into a mesh-type 3D structure consisting of spheres representing each coordinate, and cylinders connecting them [15]. Finally, using commercially available 3D modeling software (Rhinoceros 3D, version 6.0, Robert McNeel & Associates, Washington, DC, USA), the data was exported as a Standard Template Library (STL) file for 3D printing (Figure 2 and Video S2 in the supplement).

### 2.4. Thyroid 3D model Printing

All STL files were printed using a two-color 3D-fused deposition modeling printer (3DWOX 2X; Sindoh, Seoul, Republic of Korea) by a single engineer (SY). The two-color printer expressed the thyroid gland and the tumor with different colors. For evaluating the 3D printing process, the printing time including the time it took for a technician to download an STL file, transfer the file to the 3D printer, print the model, and remove supporting materials from the printed model was obtained. The cost was calculated by measuring the amount of filament used, excluding labor costs.

### 2.5. Prospective Study on Informed Consent

The main purpose of the study was to compare the degree of a patient’s understanding of their disease and surgery and their satisfaction with the informed consent with or without the use of a personalized 3D thyroid model. The conventional method of explaining consent for thyroid surgery included an illustration and standardized anatomical models (Figure S1 in the Supplement).

**Figure 3:**
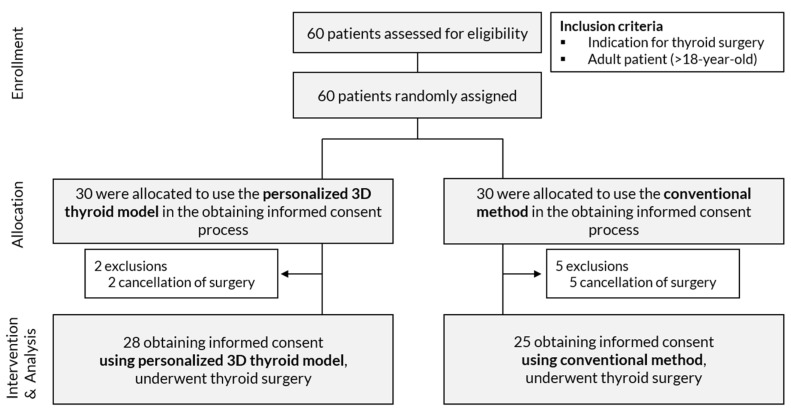
Flow chart of the prospective study.

The eligible patients were all adults with an indication for thyroid surgery. From July to October 2020, a total of 60 adult patients who had any indication for thyroid surgery were consecutively enrolled (Figure 3). After enrollment, the patients were allocated into two study arms using block-randomization rules, with a block size of four. Randomization list was created by a researcher (JS) using R software and kept by an engineer (SY) who did not face the patient directly. A personalized 3D thyroid model was prepared for each patient before obtaining informed consent regardless of their group allocation. The exclusion criteria included the withdrawal of a patient from the study or a canceled surgery between the time of obtaining informed consent for the study and the time for the thyroid surgery.

On a patient visiting day for obtaining informed consent, the attending physician was notified of the group in which the patient was enrolled, and the procedure with the 3D thyroid model was explained to the patient if they were in the 3D-printed model group. The questionnaire for informed consent (Table 1) was modified from that of Yoon et al. [11]. It was composed of four main categories (general knowledge, benefits and risks of surgery, and satisfaction). Each category consisted of three questions and each question was scored from 1 to 5, corresponding to strongly disagree, disagree, neutral, agree, and strongly agree, respectively

After surgery, all patients were provided with their 3D thyroid models and requested to complete a second questionnaire which was a shortened version of the first questionnaire. Patients rated how much their understanding and satisfaction were improved or hindered by seeing their 3D-printed model, from 1 to 5 (strongly disagree, disagree, neutral, agree, and strongly agree). If there were multiple highest ratings of improvement among the four questions, the patients were instructed to choose one of them (Table 2).

Basic information on age, sex, diagnosis, TNM classification, and operation type were collected. Information on social and past medical history was assessed at the time of admission. All patients were operated on by one of four thyroid surgeons (JS, CHR, YSJ, and JR) who shared a common policy on surgical indication and surgical extent.

All patients received ultrasonography (USG) and CT scans, which are routinely conducted in our institution preoperatively. As all DICOM files have information on slice thickness and pixel spacing, patients who had CT scans performed at other institutions using a different protocol did not require an additional CT scan.

### 2.6. Prospective Study on Informed Consent

Statistical analysis was performed using R statistical software (version 3.5.1; R Foundation for Statistical Computing, Vienna, Austria). The sample size was calculated using ‘pwr’ package, to design to detect an absolute difference of 20% between groups for the improvement or decrease of scores with 80% power, while the type I error (2-sided) was set at 5%, both groups were assumed to have a standard deviation of 25% of the mean value, and 15% drop out rate were considered. Finally, we decided to allocate 30 patients to each group. The average score for each questionnaire item was compared using Welch’s *t*-test because the two groups had unequal final sample sizes.

## 3. Results

In the process of training, our DNN model achieved the highest validation DSCs at 625th Epoch (0.932 in the training set, 0.944 in the validation set, and 0.894 in the test set) (Figure S2 in the Supplement). The DNN model at this point was loaded into our GUI application for 3D thyroid model reconstruction.

Of the 60 enrolled patients, seven patients were excluded due to a delay or cancellation of surgery. The number of patients included in the study consisted of 28 patients that provided informed consent with the use of a personalized 3D-printed model and 25 that provided informed consent with conventional tools (Table 3). Age, sex, diagnosis, pathologic diagnosis, pathologic T (pT) and N (pN) classification, and operation methods were not significantly different between the two groups. Three of the 53 patients had CT scans performed at other institutions. The social status and medical history of the enrolled patients are shown in Table S1 in the Supplement.

**Figure 4:**
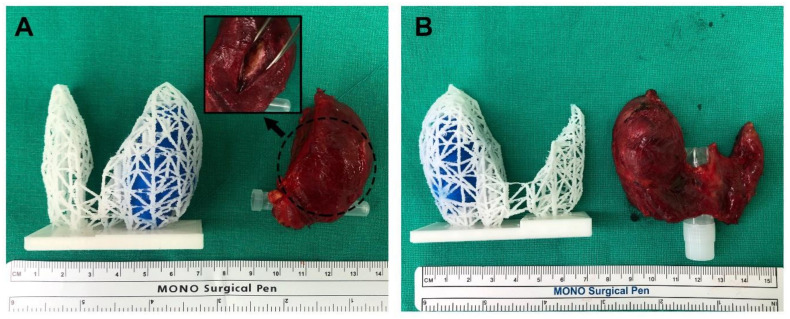
Comparison of the 3D models and actual surgical specimens (**A**,**B**). As shown in the 3D models, both cases are intraglandular tumors, similar in size and shape of the thyroid gland.

**Figure 5:**
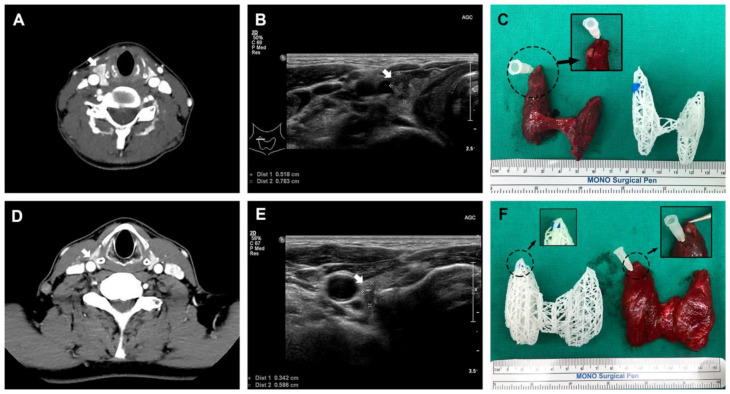
Two total thyroidectomized cases, which were clinically cT3bN1b. Each case had anterior (**A**–**C**) and posterior (**D**–**F**) external thyroidal extension into the upper pole on preoperative CT and ultrasonography, respectively (white arrow indicates thyroid cancer).

All 3D-printed models were generated without any issues. The 3D models were compared to the actual surgical specimen, and they all matched the original specimens in approximate size and the location of the lesions (Figure 4). For two example cases from patients with cT3bN1b that underwent total thyroidectomy, one was suspected to be an anterior external thyroidal extension (ETE), and the other was a posterior ETE in the upper pole based on preoperative CT and USG. The 3D models reflected each ETE from the preoperative evaluation. Surgery confirmed them as intraglandular tumors, however, the location and size of the lesions were consistent between surgical specimens and 3D-printed models (Figure 5).

### 3.1. Thyroid Gland Segmentation Using the Deep Learning Technique

For the 53 CT series from the enrolled patients, the average DSC was 0.976, which was higher than that obtained from the DNN training set (training set 0.932, validation set 0.944, and test set 0.894) (Table S2 in the Supplement). Example images of how the DNN inferred the thyroid area are presented in Figures S3–S6 in the Supplement. After segmentation, the mean post-processing time to retouch the images using the application was 4.0 min (standard deviation (SD): 3.7, min to max: 1–18), and the mean total 3D printing time was 258.9 min (SD: 78.8, min to max: 139–439). The mean price for producing the 3D-printed models was USD 4.23 (SD: 1.71, min to max: 2.15–8.88).

### 3.2. The Results of the First Questionnaire

**Figure 6:**
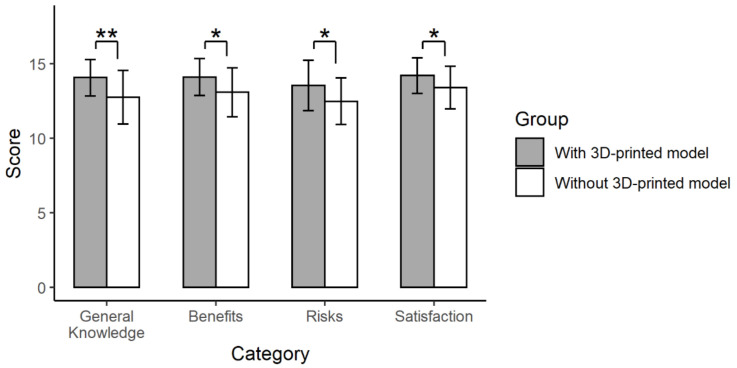
The results of the first questionnaire. Twelve items were grouped into four categories (general knowledge, benefits and risks of surgery, and satisfaction) (**, *p* < 0.01; *, *p* < 0.05).

The first questionnaire was given to patients immediately after obtaining informed consent. As a result, the ratings for 8 of the 12 items were significantly improved when using personalized 3D-printed models rather than only the conventional explanation (Table S3 in the Supplement). After dividing the questionnaire into four major categories (general knowledge, benefits and risks of surgery, and satisfaction), the group presented with 3D-printed models had significantly higher ratings in all categories than the group provided with the conventional explanation (14.1 ± 1.2 vs. 12.8 ± 1.8 for the general knowledge, *p* = 0.005; 14.1 ± 1.2 vs. 13.1 ± 1.6 for benefits, *p* = 0.016; 13.5 ± 1.7 vs. 12.5 ± 1.6 for risks, *p* = 0.026; 14.2 ± 1.2 vs. 13.4 ± 1.4 for satisfaction, *p* = 0.036) (Figure 6).

### 3.3. The Results of the Second Questionnaire

**Figure 7:**
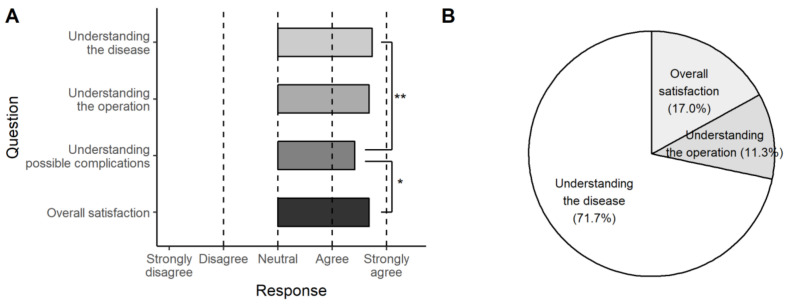
The results of the second questionnaire. The 3D-printed model was helpful in all categories (**A**) and most effective in understanding the disease (71.7%) (**B**) (**, *p* < 0.01; *, *p* < 0.05).

After surgery and receiving their 3D-printed thyroid model, none of the patients indicated that their understanding or satisfaction were worsened by the 3D-printed model. The results for all four questions placed between ‘agree’ and ‘strongly agree’; the improvement in understanding the disease and overall satisfaction were significantly higher than that of understanding the possible complications (*p* = 0.010 and 0.044, respectively) (Figure 7A). For the item with the highest improvement for individual patients, 38 patients (71.7%) chose ‘understanding the disease,’ nine (17.0%) chose ‘overall satisfaction,’ and six (11.3%) chose ‘understanding the operation’ (Figure 7B).

## 4. Discussion

The surgical procedures for thyroid disease are relatively standardized (i.e., lobectomy or total thyroidectomy). However, the extent of surgery is decided by a comprehensive assessment, including tumor size (micro-or macro-), location (lobe or isthmus), invasion into surrounding tissue, and patient preference [17]. In other words, when the need for lobectomy or total thyroidectomy is not conclusive, a patient’s preference could decide the approach. Therefore, providing patients with sufficient knowledge of their disease may be the most important part of the surgical decision. In this regard, the thyroid gland may be an appropriate organ to implement patient-specific 3D-printed models for enhancing a patient’s understanding of their disease and treatment.

Although 3D printing has already been integrated into current medical practice [9], one of the greatest limitations is the cost of producing the 3D-printed model [18]. Another limitation is the modeling and printing time. In a study on a 3D-printed replica model of the pulmonary artery, the design process took eight hours, and the printing took 97 h [19]. Therefore, although many studies have been done on methodologies for patient-specific (i.e., personalized) modeling according to various anatomical sites or different purposes such as surgical guide or medical training [20,21,22,23,24], their usefulness in the clinical setting has only been demonstrated in a few studies [10,11,14,25].

In these regards, the strength of the study was that we demonstrated that personalized 3D models could be successfully created by using deep learning segmentation and a mesh-type 3D modeling technique with reduced time and cost. Furthermore, the personalized 3D-printed model was proven to improve a patient’s understanding and satisfaction. We also demonstrated that we could successfully integrate our trained DNN into a user-friendly application. The application allowed the direct updating of the inferred result, thereby reducing the time required to prepare more training datasets to improve the performance of the DNN.

### 4.1. Fabrication of Personalized Thyroid 3D-Printed Models

We chose U-Net [16], a widely used deep learning architecture for semantic segmentation, as a backbone. Although our dataset consisted of only 106 CT series, we achieved approximately 0.9 of DSCs by using image augmentation in the process of training. Interestingly, our DNN outperformed in the prospective study compared to the training process. The reason was that the DSC while training was calculated using the annotated area which was determined by a physician without any prior information as the ground truth. In contrast, the DSC for the prospective study was calculated using the post-processed segmentation area as the ground truth, however, the post-processing was performed after the physician referred to the inference of the DNN. As each anatomical structure in the CT or MRI image may have low contrast of adjacent organs [26,27], some of the errors in the inferenced segmentation may be tolerated based on the clinician’s judgment, and eventually this discrepancy might have led to better performance in the prospective study.

Using a commercially available two-color 3D printer (approximately USD 5000 in Republic of Korea), we printed all 3D models without any issues. The mean total 3D printing time was 258.9 min, and the mean price for production was USD 4.23. These results indicated that a 3D-printed model could be produced at a reasonable price and used on the same day once a CT was taken. However, the cost-effectiveness of our model could not be easily assessed because the quality, size, and number of colors used in 3D printing were different among different studies, and it was difficult to find a study that presented data on both cost and time. For two studies using high-quality and detailed 3D-printed models, the cost was hundreds of dollars, but the printing time was not reported [10,11]. Another study on lumbar spinal disease reported that its price per model was USD 30–45, but no printing time was provided [28]. In contrast, a study on articular fracture reported a printing time of 6–12 h, but the cost was not presented [25].

### 4.2. The Usefulness of Personalized 3D-Printed Thyroid Models

To evaluate the usefulness of the personalized 3D-printed thyroid models, we used two similar questionnaires. The first questionnaire consisted of 12 items to compare two randomized groups of patients at the time of obtaining informed consent. The second questionnaire consisted of four items representing four categories and was provided to patients after surgery and receiving their model.

For the first questionnaire, there were no significant differences between the two randomly assigned groups in terms of age, sex, diagnosis, pathological classification, and operation type (all *p* > 0.05). When the 12 items of the first questionnaire were grouped into four major categories (general knowledge, benefits and risks of surgery, and satisfaction), the 3D-printed models significantly improved the scores in all categories (all *p* < 0.05). These results differed from those of a similar study on lung cancer, where only the knowledge category was significantly higher in the group receiving 3D-printed models [11]. This conflicting result may be due to their smaller number of enrolled patients compared to our study. Another prospective study on thyroid cancer showed significant differences in anatomy, the relation of structures, and surgical procedures before and after an explanation using a common 3D phantom model [14]; however, it was not a randomized study and did not use individualized models. 

With the second questionnaire, we found that patients found the greatest value in ‘understanding the disease’ (71.7%), followed by ‘overall satisfaction’ (17.0%) and ‘understanding the operation’ (11.3%). These data suggest that the mesh-type 3D modeling technique might have value for anatomical education, even though the models were not of high quality. The presentation of the location relationship between the tumor and thyroid gland may be the biggest advantage of this type of 3D-printed model.

### 4.3. Limitations

There are several limitations to this study. Although the patients were randomly assigned to the two study groups, the clinician’s explanations of consent for surgery could differ because the study was not double-blinded. In addition, the ‘understanding’ of the patients was not evaluated objectively.

CT may not be a routine procedure for all thyroid surgeries. In our country, preoperative CT is covered by national insurance and is acceptable for the purposes of surgical planning, evaluation of regional metastasis, and preventing legal disputes, however, it may be unnecessary to perform CT scans for 3D printing in a different clinical setting.

Another important limitation is that incorporation of 3D printed models into patient’s treatment could not be assessed, such as how it improved treatment outcomes or patient’s care. It is necessary to expand the scope of application of the 3D model for each disease stage and treatment.

Finally, to reduce costs and time, our 3D-printed models were not of high quality. Thin section CT scans (e.g., 1 mm splices) could provide more detailed information [29,30], however, it could be harmful to patients by exposing them unnecessarily to additional radiation doses. From the results of this study, the specific purpose of educating a patient on thyroid surgery can be achieved even under conditions where only the minimum necessary information is depicted. Indeed, the approximate size of the thyroid glands and the locations of the tumors were matched when compared to the surgical specimens.

## 5. Conclusions

We established the methodology of personalized 3D-printed thyroid models using U-Net based deep learning and the mesh-type 3D-modeling techniques. In addition, we confirmed the usefulness of personalized 3D-printed thyroid models in obtaining informed consent. The 3D-printed model could improve a patient’s understanding of the disease and surgery and overall satisfaction. Although the quality of the mesh-type 3D-printed models was not realistic, they were effective in educating the patients on the anatomy of the disease by showing the anatomical relationship between the tumor and thyroid gland and improving overall patient satisfaction.

## 6. Patent

Republic of Korea Patent No. 10-2227735, filed 3 January 2020, and issued 9 March 2021.

## Figures and Tables

**Table 1:** First questionnaire administered immediately after obtaining informed consent with or without personalized 3D-printed thyroid model for thyroidectomy in the outpatient clinic.

Questions ^1^
**General Knowledge**
I understand the location and size of thyroid lesion that will be surgically resected I understand the disease status of the thyroid lesion(s) (malignant/benign) I understand the surgical procedure that will be performed
**Benefits**
4. I understand the purpose of the surgery 5. I understand the extent of the surgery and its rationale 6. I understand the expected survival and recurrence rates after treatment
**Risks**
7. I understand the potential complications related to the surgery 8. I understand the possibility of a delayed discharge from the hospital due to complications or the need for additional treatment 9. I understand that the potential complications may vary depending on the location and size of the lesion in relation to recurrent laryngeal nerve and blood vessels
**Satisfaction**
10. I am sufficiently informed about the surgery and satisfied with the explanation 11. I am satisfied with my decision and the medical staff 12. I am satisfied with my understanding of the disease

^1^ Rating: (1) strongly disagree, (2) disagree, (3) neutral, (4) agree, and (5) strongly agree.

**Table 2:** Second questionnaire administered immediately after providing the personalized 3D-printed thyroid model.

Question ^1^
The personalized 3D-printed thyroid model enhanced my understanding of my thyroid disease (cancer/nodule). The personalized 3D-printed thyroid model enhanced my understanding of the reason, extent, and procedure of the surgery. The personalized 3D-printed thyroid model enhanced my understanding of the possible complications and adverse results of the surgery. The personalized 3D-printed thyroid model enhanced my satisfaction with the clinicians and institution.
- If the highest degree of improvement was more than two, which of the items do you think is the most helpful? (Items 1–4)

^1^ Rating: (1) strongly disagree, (2) disagree, (3) neutral, (4) agree, and (5) strongly agree.

**Table 3:** Demographics of the enrolled patients.

		With 3D Model (n = 28)	With Conventional Tools (n = 25)	*p*
Age		49.2 ± 11.3	42.4 ± 15.8	0.083 *
Sex	Female	24 (85.7%)	18 (72.0%)	0.313 ^†^
	Male	4 (14.3%)	7 (28.0%)	
Pathologic diagnosis	Malignant ^§^	25 (89.3%)	25 (100.0%)	0.238 ^†^
	Benign	3 (10.7%)	0 (0.0%)	
pT classification ^‡^	1	21 (84.0%)	19 (76.0%)	0.615 ^†^
	2	0 (0.0%)	2 (8.0%)	
	3	4 (16.0%)	4 (16.0%)	
	4	0 (0.0%)	0 (0.0%)	
pN classification ^‡^	0	13 (52.0%)	15 (60.0%)	0.639 ^†^
	1a	6 (24.0%)	7 (28.0%)	
	1b	6 (24.0%)	3 (12.0%)	
Operation	Lobectomy	18 (64.3%)	13 (52.0%)	0.413 ^†^
	Total thyroidectomy	10 (35.7%)	12 (48.0%)	

Abbreviations: pT, pathological T; pN, pathological N; * Welch’s *t*-test; **^†^** Fisher’s exact test; ^‡^ The number of pT and pN classifications were only for malignant cases; ^§^ One case of follicular thyroid carcinoma was included in the group without 3D model; otherwise, the tumors were papillary thyroid carcinoma.

## Data Availability

The authors confirm that the data supporting the findings of this study are available within the Supplementary Materials.

## Supplementary Materials

The following are available online at https://www.mdpi.com/article/10.3390/jpm11060574/s1, Video S1. The process of our application for deep learning segmentation, modification, marking of thyroid nodules, and exporting the model to mesh-type 3D modeling data. Video S2. Loading of 3D modeling data and its export to a Standard Template Library (STL) file using commercially available 3D modeling software. Figure S1. Standardized anatomical models (a) and an illustration of the thyroid anatomy (b). Figure S2. Change in the Dice similarity coefficient (DSC) according to deep learning model training (a) and change of loss value defined as −1 * DSC (b). Table S1. Social status and medical history of the enrolled patients (n = 53). Table S2. Log data for 53 patients enrolled in the study. Figure S3. Original Thyroid CT images (slices 72–91) for patient S004. Figure S4. Segmentation results for the CT slices of patient S004. The blue mask is the area segmented by the deep learning model. The Dice similarity coefficient was 1.000 and required no correction. Segmentation was successfully achieved in slices where the thyroid gland was observed. Figure S5. Original Thyroid CT images (slices 62–85) for patient S007. The patient has an enlarged left thyroid. Figure S6. Segmentation results for the CT slices of patient S007. The blue mask is the area segmented by the deep learning model. The Dice similarity coefficient was 0.790 and recorded the lowest accuracy. From 76th slide, the goitrous left thyroid lobe was not completely segmented. Table S3. Detailed results of the first questionnaire.
