# Supplementary material for: A Personalized 3D-Printed Model for Obtaining Informed Consent Process for Thyroid Surgery: A Randomized Clinical Study Using a Deep Learning Approach with Mesh-Type 3D Modeling
Source: J Pers Med. 2021 Jun 18;11(6):574. doi: 10.3390/jpm11060574 (PMC8234549; doi:10.3390/jpm11060574)
Supplement: Supplementary file 1 [file jpm-11-00574-s001.zip › Supplement materials_JPM_final.pdf]

Supplementary videos

Video S1. The process of our application for deep learning segmentation, modification, marking of thyroid nodules, and exporting the model to mesh-type 3D modeling data.

Video S2. Loading of 3D modeling data and its export to a Standard Template Library (STL) file using commercially available 3D modeling software.

Supplementary Figures

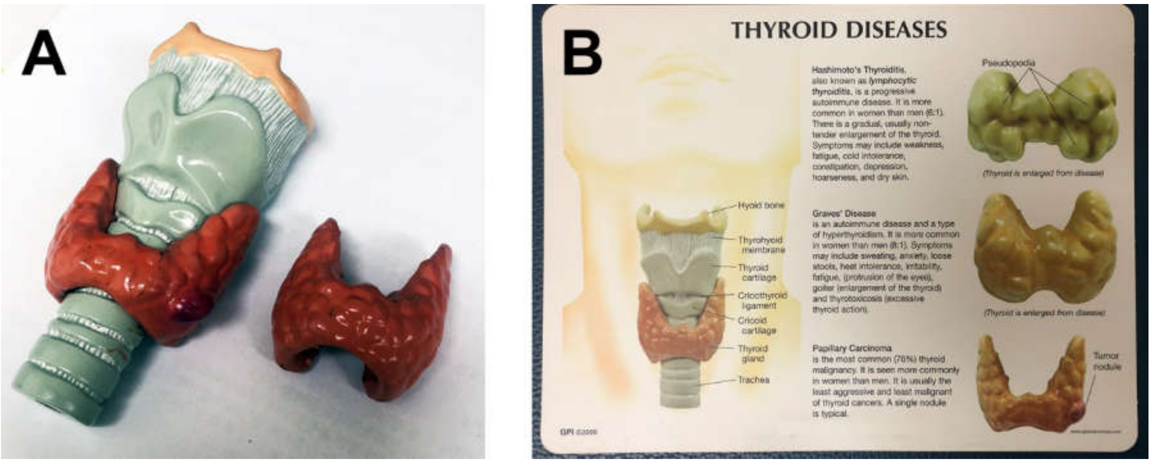

Figure S1. Standardized anatomical models (**A**) and an illustration of the thyroid anatomy (**B**).

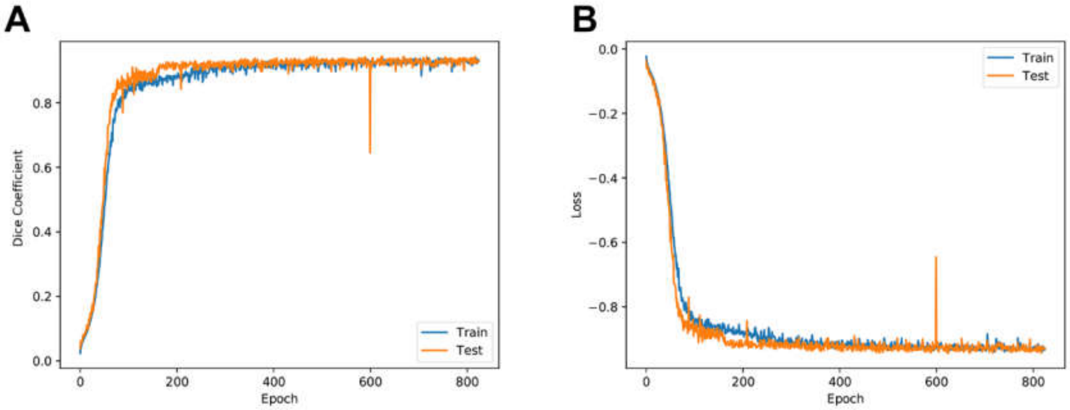

Figure S2. Change in the Dice similarity coefficient (DSC) according to deep learning model training (**A**) and change of loss value defined as *-1 * DSC* (**B**).

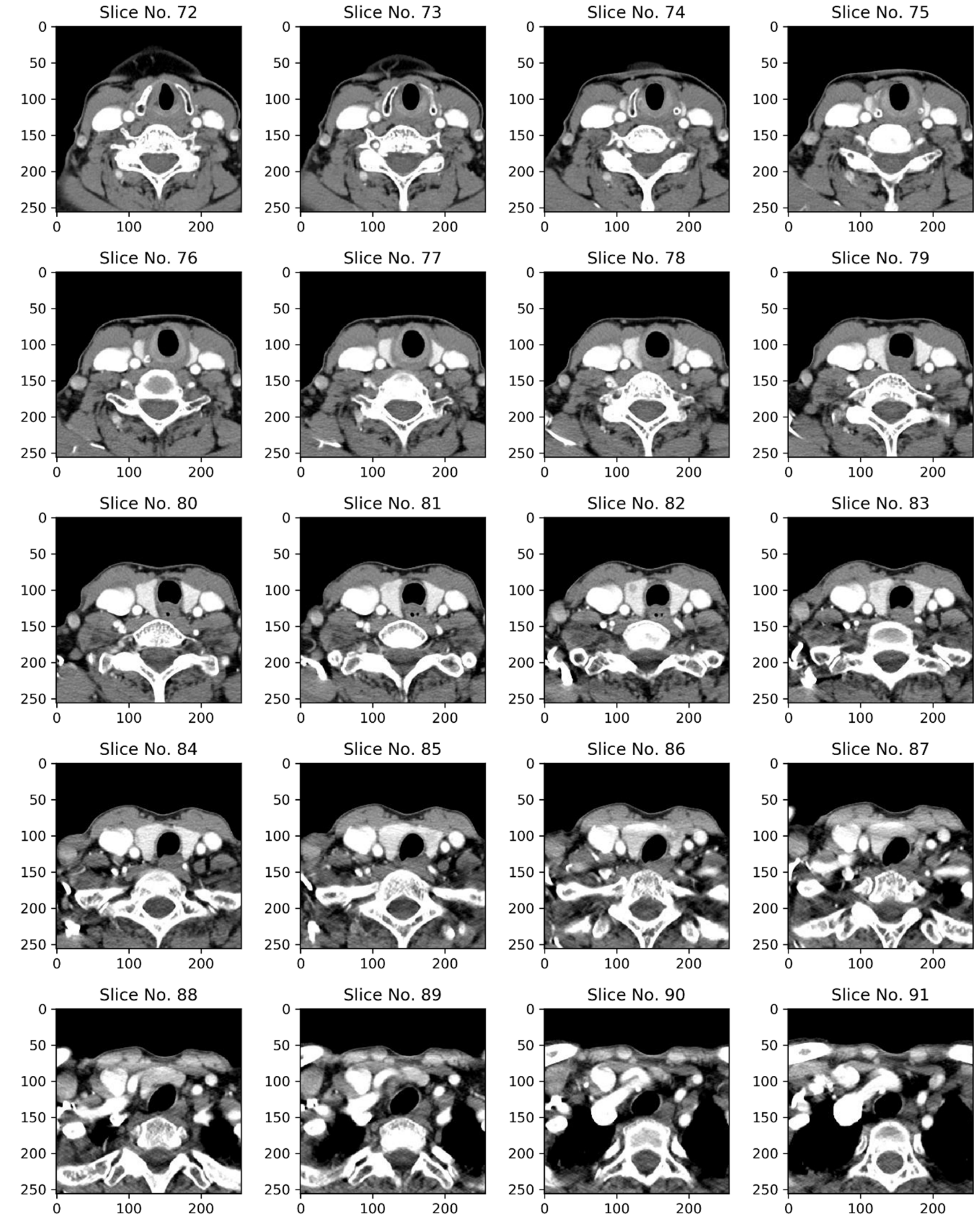

Figure S3. Original Thyroid CT images (slices 72 to 91) for patient S004.

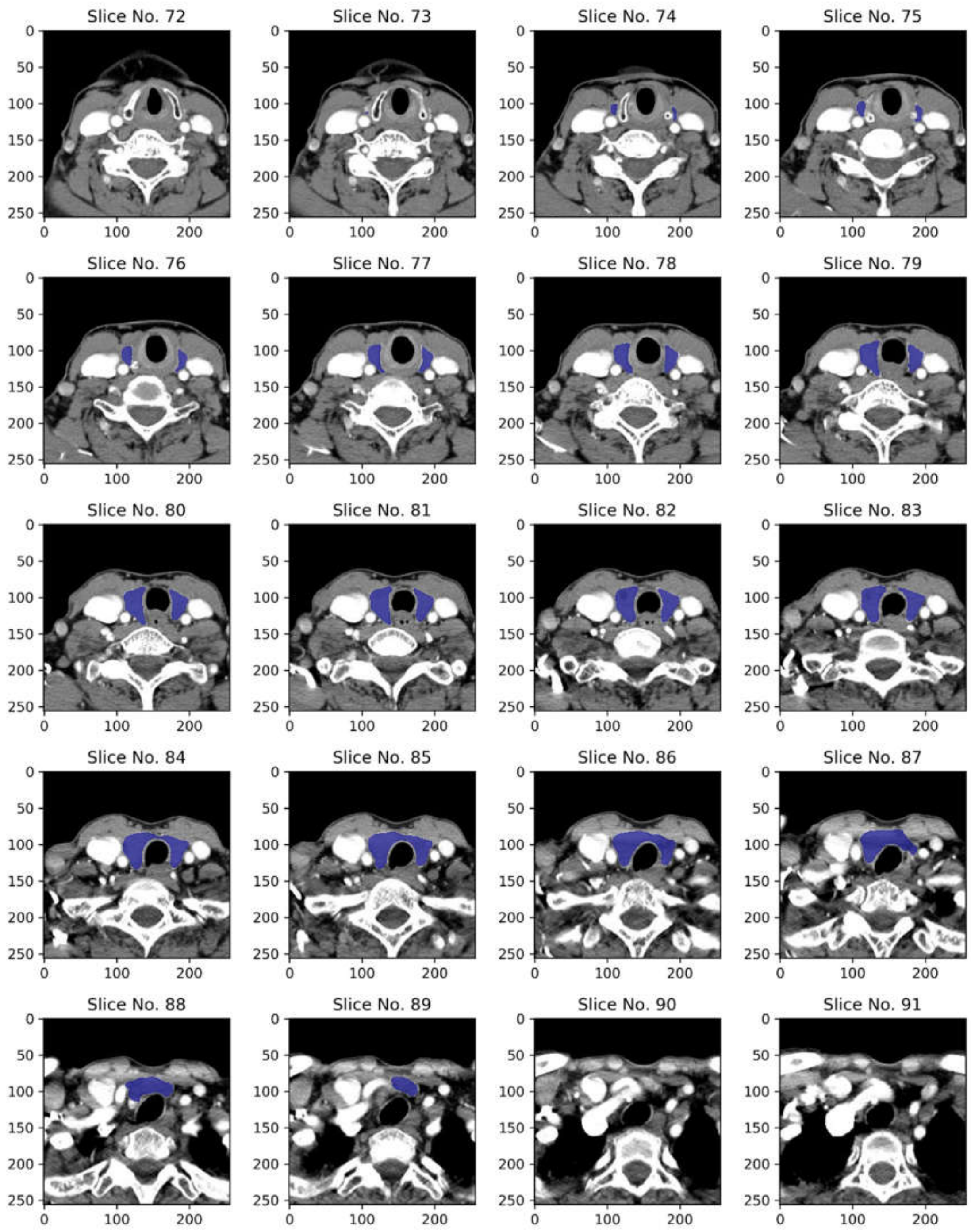

Figure S4. Segmentation results for the CT slices of patient S004. The blue mask is the area segmented by the deep learning model. The Dice similarity coefficient was 1.000 and required no correction. Segmentation was successfully achieved in slices where the thyroid gland was observed.

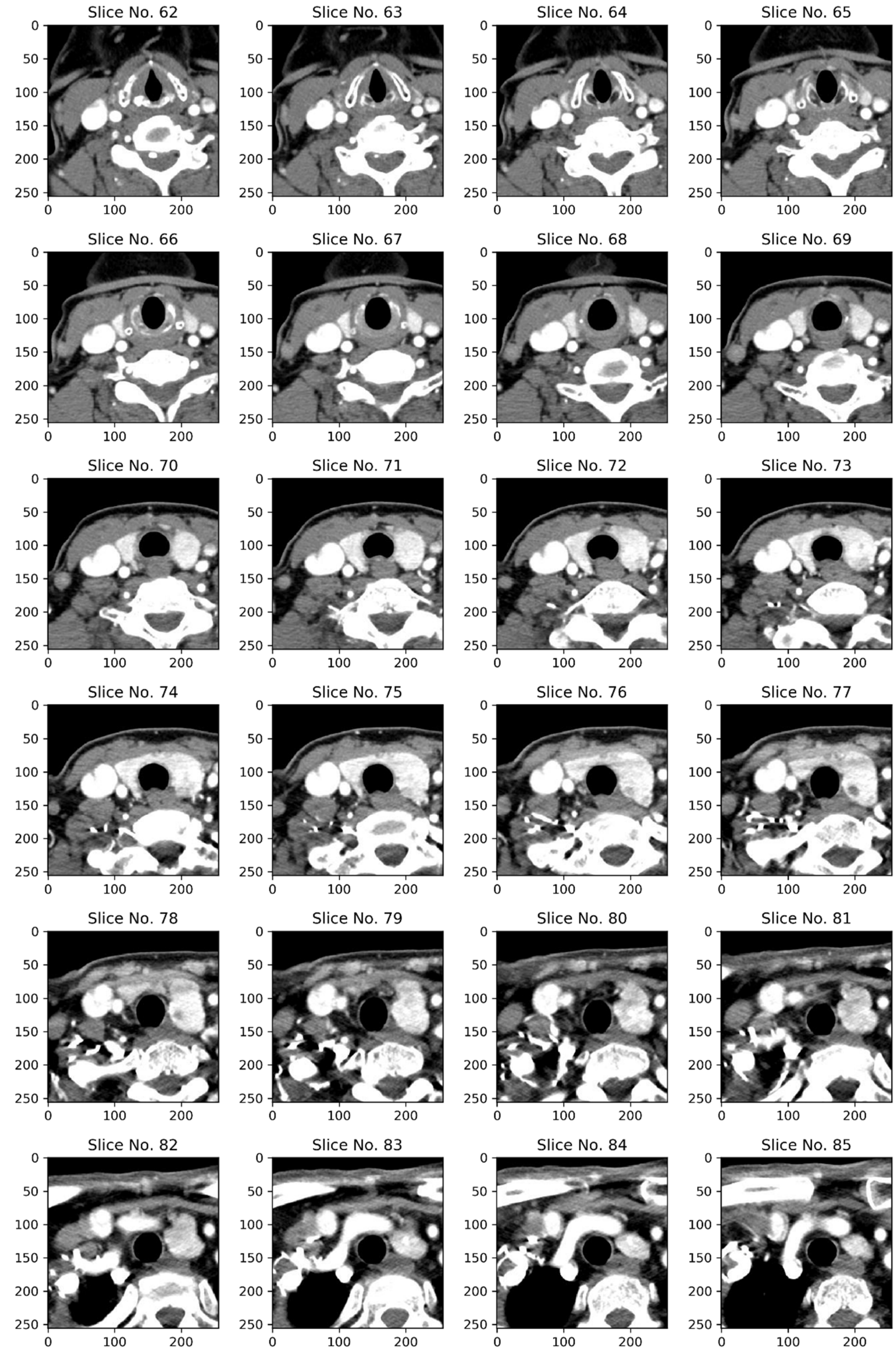

Figure S5. Original Thyroid CT images (slices 62 to 85) for patient S007. The patient has an enlarged left thyroid.

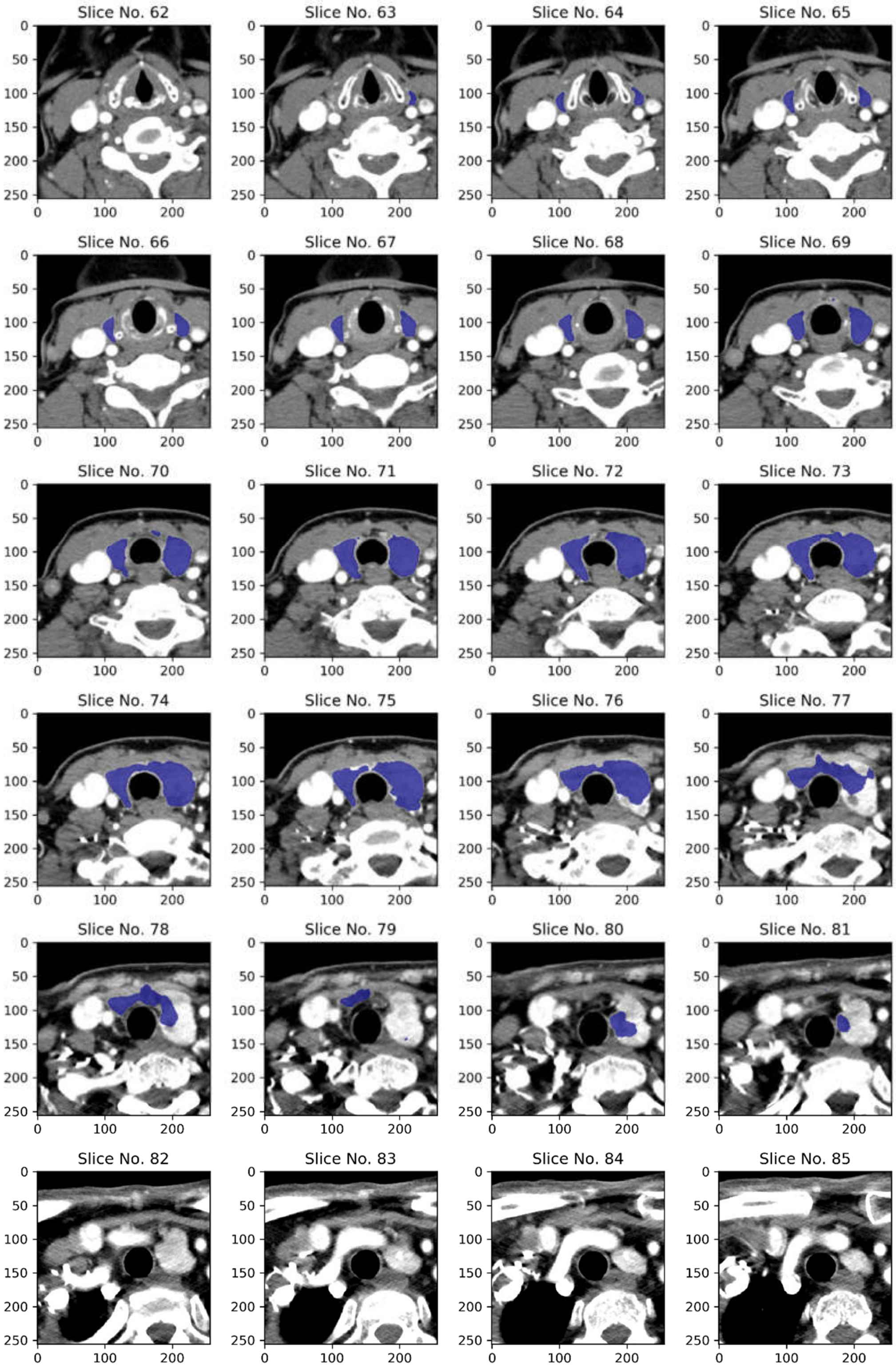

Figure S6. Segmentation results for the CT slices of patient S007. The blue mask is the area segmented by the deep learning model. The Dice similarity coefficient was 0.790 and recorded the lowest accuracy. From 76th slide, the goitrous left thyroid lobe was not completely segmented.

Supplementary Tables

Table S1. Social status and medical history of the enrolled patients (n = 53)

Cohabitation status	
Cohabitant	45 (84.9%)
Alone	8 (15.1%)
Education	
University or higher graduate	28 (52.8%)
Secondary school or lower graduate	25 (47.2%)
Employment	
Employed	35 (66.0%)
Unemployed	17 (32.1%)
Full-time student	1 (1.9%)
Observe religion	
Yes	21 (39.6%)
No	32 (60.4%)
Smoking	
Active	1 (1.9%)
Ex-smoker	6 (11.3%)
Never	46 (86.8%)
Physical status	
ASA ≤ 2	53 (100.0%)
ASA ≥ 3	0 (0.0%)
Family history of thyroid cancer	
Yes	6 (11.3%)
No	47 (88.7%)
Previous surgery, including surgery with local anesthesia	
Yes	36 (69.8%)
No	17 (30.2%)

Table S2. Log data for 53 patients enrolled in the study

Serial No.	CT protocol	Dice coefficient	Post processing time after segmentation (min)	3D printing time (min)	Support material removal time (min)	Price ($)
S003	Thyroid CT (contrast)	0.999	2	134	5	2.44
S004	Thyroid CT (contrast)	1.000	2	181	10	3.41
S007	Thyroid CT (contrast)	0.790	10	330	5	5.17
S008	Thyroid CT (contrast)	0.993	4	225	5	5.17
S009	Thyroid CT (contrast)	0.966	3	186	5	3.90
S010	Thyroid CT (contrast)	0.967	6	206	5	4.39
S012	Thyroid CT (contrast)	0.986	3	177	5	2.15
S013	Thyroid CT (contrast)	0.981	3	303	5	7.41
S014	Thyroid CT (contrast)	0.922	6	151	5	2.83
S015	Thyroid CT (contrast)	0.910	18	393	5	6.73
S016	Thyroid CT (contrast)	0.993	3	174	5	3.32
S017	Thyroid CT (contrast)	0.985	4	237	5	3.32
S019	Thyroid CT (contrast)	0.968	3	220	5	3.80
S020	Thyroid CT (contrast)	0.894	18	388	5	7.90
S021	Thyroid CT (contrast)	0.983	3	230	5	2.93
S022	Thyroid CT (contrast)	0.966	3	151	5	3.22
S023	Thyroid CT (contrast)	0.982	3	158	5	3.02
S024	Thyroid CT (contrast)	0.991	4	220	5	4.19
S025	Thyroid CT (contrast)	0.948	5	266	5	4.00
S026	Thyroid CT (contrast)	0.997	2	213	5	2.73
S027	Thyroid CT (contrast)	0.980	4	215	5	4.09
S028	Thyroid CT (contrast)	0.991	3	200	5	2.34
S029	Thyroid CT (contrast)	0.994	2	249	5	3.51
S030	Thyroid CT (contrast)	0.997	2	214	5	4.49
S031	Thyroid CT (contrast)	0.998	2	220	5	5.17
S032	Thyroid CT (contrast)	0.968	4	287	5	3.61
S033	Thyroid CT (contrast)	0.999	2	150	5	2.73
S034	Thyroid CT (contrast)	0.984	2	275	5	4.29
S035	Thyroid CT (contrast)	0.941	6	284	5	5.27
S036	Thyroid CT (contrast)	0.996	2	202	5	2.34
S037	Thyroid CT (contrast)	0.993	2	231	5	3.12
S038	Thyroid CT (contrast)	0.996	3	223	5	2.93
S039	Thyroid CT (contrast)	0.961	6	424	15	7.51
S040	Thyroid CT (contrast)	0.989	3	225	5	2.73
S041	Thyroid CT (contrast)	0.985	2	410	10	4.29
S042	Thyroid CT (contrast)	0.995	2	332	5	4.88
S043	Thyroid CT (contrast)	1.000	1	231	5	2.63
S044	Thyroid CT (contrast)	0.995	3	236	5	2.93
S045	Thyroid CT (contrast)	0.987	2	304	5	5.27
S046	Thyroid CT (contrast)	1.000	2	189	5	3.61
S047	Outside (contrast)	0.965	4	335	5	5.17
S048	Outside (contrast)	0.889	11	420	10	8.39
S049	Outside (contrast)	0.993	4	282	5	3.61
S050	Thyroid CT (contrast)	0.998	2	200	5	2.15
S051	Thyroid CT (contrast)	1.000	1	258	5	2.83
S052	Thyroid CT (contrast)	0.985	3	265	5	4.10
S054	Thyroid CT (contrast)	0.994	3	342	5	5.17
S055	Thyroid CT (contrast)	0.993	3	395	10	7.02
S056	Thyroid CT (contrast)	1.000	2	218	5	3.61
S057	Thyroid CT (contrast)	0.927	14	188	5	8.88
S058	Thyroid CT (contrast)	0.994	3	256	5	3.71
S059	Thyroid CT (contrast)	0.998	3	423	5	7.32
S060	Thyroid CT (contrast)	1.000	1	203	5	2.63

Table S3. Detailed results of the first questionnaire

	With 3D-printed model group (n = 28)	With conventional method group (n = 25)	*p**
**General Knowledge**			
1. I understand the location and size of thyroid lesion that will be surgically resected	4.6 ± 0.6	4.3 ± 0.6	0.095
2. I understand the disease status of the thyroid lesion(s) (malignant/benign)	4.8 ± 0.4	4.3 ± 0.6	0.002
3. I understand the surgical procedure that will be performed	4.7 ± 0.5	4.2 ± 0.7	0.007
**Benefit**			
4. I understand the purpose of the surgery	4.6 ± 0.6	4.5 ± 0.5	0.234
5. I understand the extent of the surgery and its rationale	4.8 ± 0.4	4.4 ± 0.6	0.004
6. I understand the expected survival and recurrence rates after treatment	4.6 ± 0.6	4.2 ± 0.7	0.026
**Risk**			
7. I understand the potential complications related to the surgery	4.5 ± 0.6	4.0 ± 0.6	0.006
8. I understand the possibility of a delayed discharge from the hospital due to complications or the need for additional treatment	4.4 ± 0.7	4.3 ± 0.6	0.332
9. I understand that the potential complications may vary depending on the location and size of the lesion in relation to recurrent laryngeal nerve and blood vessels	4.5 ± 0.6	4.1 ± 0.6	0.018
**Satisfaction**			
10. I am sufficiently informed about the surgery and satisfied with the explanation	4.7 ± 0.4	4.5 ± 0.5	0.057
11. I am satisfied with my decision and the medical staff	4.7 ± 0.6	4.5 ± 0.5	0.142
12. I am satisfied with my understanding of the disease	4.8 ± 0.4	4.4 ± 0.5	0.014

*, Wilcoxon rank sum test
